# The usefulness of pre-employment and pre-deployment psychological screening for disaster relief workers: a systematic review

**DOI:** 10.1186/s12888-020-02593-1

**Published:** 2020-05-11

**Authors:** Elena Opie, Samantha Brooks, Neil Greenberg, G. James Rubin

**Affiliations:** NIHR Health Protection Research Unit in Emergency Preparedness and Response, King’s College London, Weston Education Centre, Cutcombe Rd, London, SE5 9RJ UK

**Keywords:** Resilience factors, Predictors, Disaster relief workers, Psychological distress, Psychological disorder

## Abstract

**Background:**

Individuals who conduct disaster relief work overseas are exposed to a variety of traumatic events that can cause distress and trigger psychological illnesses. Identification of which disaster relief workers may be at risk of experiencing psychological distress or mental health disorders is frequently carried out through pre-employment or pre-deployment psychological screening. The primary objective of our review was to assess the evidence for pre-employment and pre-deployment psychological screening of relief workers who work in disaster situations. We aimed to identify specific pre-employment and pre-deployment characteristics that predict impaired wellbeing of an individual following engaging in disaster-related work.

**Methods:**

A combined list of search terms was composed relating to disaster-related occupations, screening methods, psychological disorders, and study design. The databases used were PsycINFO, MEDLINE, EMBASE, and GlobalHealth. We included studies that used cross-sectional or longitudinal study designs; were published in the English language in peer-reviewed academic journals; reported on the association between pre-employment and pre-deployment features and post-deployment psychological disorders or distress; considered any occupational groups responding to a specified, discrete crisis; and used at least one validated measure of distress or disorder. We extracted data on the author; year of publication; disaster description; country of study; study design; population sample; disorder(s) outcome and the measures used; and results.

**Results:**

Sixty-two, high-quality studies were included in the review. Forty-one potential predictors were identified. Of these, only volunteer status and previous history of mental illness and life stressors emerged as reliable predictors of distress or disorder.

**Conclusion:**

The results suggest that whilst it is attractive to screen for pre-employment and pre-deployment indicators of resilience, the evidence base for doing so is weak. At best, this sort of screening can only weakly suggest vulnerability and at worst may result in discrimination. Until better evidence about its usefulness becomes available, employers should exercise caution over its use.

## Background

Individuals who conduct disaster relief work overseas may be exposed to traumatic events that can cause distress and trigger psychological illnesses such as post-traumatic stress disorder (PTSD), depression and anxiety disorders [1–6]. Disaster relief workers include professionals such as firefighters, police, search-and-rescue and relief teams, and medical personnel alongside volunteer relief workers with no formal training. Previous research has identified that the rates of PTSD amongst these populations range from 8 to 25% [7–9]. Whilst these figures underline the risk associated with relief work, they also indicate that only a minority of trauma-exposed workers develop psychological distress and illness [10]. This raises the question of whether it is possible to predict which individuals will be susceptible to psychological distress or disorder following the trauma exposure that can occur in disaster relief work.

Identification of disaster workers who might be vulnerable to developing post-trauma mental health disorders is often attempted via pre-employment or pre-deployment psychological screening methods. Those identified as being vulnerable may be prevented from taking up a disaster worker role, be more closely monitored during their work or be restricted in the duties they can undertake. However, pre-deployment psychological screening has a chequered history. In World War II, the USA identified around 2 million men as being at risk of breaking down under the stress of combat, the majority of whom subsequently proved to be resilient, effective soldiers [11]. Recent efforts to screen military personnel have demonstrated a similar lack of precision [12]; a study [13] screened military personnel for mental disorders pre-deployment, a frequently reported risk-factor [14], only to find that it had low positive predictive value for developing mental health disorders post-deployment.

The apparent lack of effectiveness of screening tools has encouraged researchers to expand their search for risk and resilience factors in populations working in trauma prone environments. A recent review on screening within the emergency services [15] identified multiple predictors of mental health including neuroticism, pre-existing psychopathology, trauma history, maladaptive coping styles including catastrophic thinking and rumination, and social factors, such as substance related disorders. Still, the study came to conclude that there were no reliable ways to enhance personnel selection through screening and the authors deduced that further research on this topic was needed. The review [15], however, was limited to individuals working in general emergency service roles rather than disaster responders with most of the identified studies only including trainee police officers and firefighters.

The primary objective of our review is to assess the evidence for pre-employment and pre-deployment psychological screening of disaster response workers. We aimed to identify specific characteristics, which can be detected pre-engagement, that predict impaired wellbeing of an individual following their work.

## Methods

This systematic review was conducted with reference to the PRISMA criteria [16].

### Protocol and registration

An unpublished protocol was developed by the research team and was reviewed and edited by each member. The protocol was not registered.

### Study selection

We included studies if they met all the following criteria:

- Published in peer-reviewed, academic journals;
- Published in English;
- Considered any occupational group responding to a specified, discrete crisis;
- Reported on the association between pre-employment and/or pre-deployment features and post-deployment psychological disorders or distress;
- Primary, quantitative research-based longitudinal or cross-sectional study designs;
- Used at least one validated measure of distress or disorder.

Only published papers written in the English language were included in the review due to the resources available to conduct this systematic review. For the same reason, grey literature was not included, also to ensure that all studies included had gone through a peer review process. There were no limits on the year of publication or of follow-up time to include all the relevant research. Studies relating to military personnel were included if the personnel were studied in relation to a discrete humanitarian incident or disaster, but not if they were studied in relation to a combat or peacekeeping missions, since the nature of these duties would provide data that was not relevant to the research question.

### Conducting the review

A combined list of search terms was composed by examining relevant past literature (Additional file 1). The terms related to disaster-related occupations, screening methods, psychological disorders, and study design. In August 2018, one author (EO) conducted the search using the following databases: PsycINFO (1806–2018), MEDLINE (1946–2018), EMBASE (1980–2018), GlobalHealth (1966–2018). The thesaurus of each database was checked regarding each term to ensure applicability for that database. A final search was conducted by EO on the 18/10/2018 to update the review. EO also undertook thorough reference checking and tracking, including forward citation tracking, of each included study and of relevant systematic reviews. In turn, this strengthened the search and greatly reduced the risk of missing relevant papers. All resulting citations were downloaded to EndNote© software version X7 [17].

Bibliographic searching was completed in August 2018 and a total of 8426 potentially relevant papers were identified (including the papers identified via reference checking and tracking). Upon removing all duplicate studies, 5627 studies remained. All titles and abstracts were screened by EO, and the papers excluded if they did not correspond to the inclusion criteria; 227 studies remained. After reviewing the full papers, 165 papers were excluded and a total of 62 papers were included in the review. Please see Additional file 3 for the Flow Diagram of the study selection process.

The general causes of exclusion were that many of the studies were not of longitudinal or cross-sectional design, did not include completion of pre-deployment measures, were based on military samples working in combat or peacekeeping missions, were not based on a specified, discrete crisis and/or did not use at least one validated measure.

### Data extraction, quality appraisal, and data synthesis

EO was trained on how to use the data collection form by SKB [18]. EO piloted using the form with one paper, which SKB then reviewed; discussion for any areas for improvement was had. Coding instructions were clearly stated on the data collection form. Using the data collection form, all papers underwent data extraction by EO (Additional File 4. Main Results Table). A small number of these papers had data independently extracted by SKB. Upon a comparison of the extracted data by both researchers, consensus was generally high. Any disagreements were solved by discussion between the research team.

We extracted and recorded the following variables from each included study: author, year of publication; disaster description; country of study; study design; time assessed since deployment/follow-up time; population sample (‘*n*’ and demographic data including gender split, age range, mean age, job role); disorder outcome(s) and the measures used; predictive factors including key results.

We assessed the quality of each study in three areas: study design; data collection and methodology; and results. Our quality assessment tool (Additional file 2) was designed for a previous review [18] and was informed by existing quality appraisal tools [19, 20]. Each study was given an overall score as a percentage, based on the number of ‘yes’ responses, with a higher score indicating better quality.

For data synthesis, thematic analysis was used to group predictive factors into a typology. Topics we accepted as “themes” were required to be identified by at least two studies to be discussed in the in-text results; predictor variables explored by only a single study are reported only in the tables.

Thematic analysis was also used to group psychiatric distresses into a typology; in Tables 1-7, secondary trauma was re-grouped into PTSD given that the symptoms are very similar [81] and acute stress was re-grouped into stress. It should be noted that studies often reported psychological distress or morbidity as the outcome without stating whether participants were likely to have a specified mental health diagnosis or not.

**Table 1:** Demographics as a predictor of psychological distress following disaster relief work

Disorder	Papers reporting significant association with disorder / papers reporting non-significant association with disorder
PTSD	Age [7, 21–35] / [8, 36–54] Gender [23, 25, 28–30, 35, 37, 43, 53–55] / [8, 21, 22, 31, 33, 36, 38–42, 46, 48–50, 52, 56, 57] Ethnicity [22, 25, 28–30] / [23, 31, 36, 44, 49, 51, 54, 57] Education [22, 25, 28–31] / [8, 33, 34, 39, 40, 42, 44, 49, 51–54, 58] Marital status [22, 24, 27–30, 42, 51] / [7, 8, 26, 33, 34, 36, 38–40, 43, 44, 47, 53, 57] Income [28, 30] / [23, 29, 33, 49] Social class / [32] Religious group / [54]
Anxiety	Age [48, 54] / [21, 22, 47, 51] Gender [22, 54] / [21, 48] Ethnicity [22] / [51, 54] Education [22] / [51, 54] Marital status [22] / [47, 51] Religious group / [54]
Depression	Age [22, 54, 59] / [21, 39, 42, 43, 47, 48, 50–52] Gender [22, 48, 54] / [21, 39, 42, 43, 50, 52, 59] Ethnicity [22, 59] / [51, 54] Education [22, 52] / [39, 42, 51, 54, 59] Marital status [22, 42, 47] / [39, 43, 51, 59] Religious group / [54]
Psychological distress / morbidity	Age [7, 26, 41, 60] / [23, 24, 49, 61–63] Gender [23] / [41, 49, 55, 61, 63] Ethnicity / [23, 49, 63] Education / [49, 58, 63] Marital status [7] / [24, 26, 61] Income / [23, 49] Social class / [62]
Somatic symptoms	Age / [21, 47, 51, 54] Gender [54] / [21] Ethnicity [51] / [54] Education / [51, 54] Marital status [47] / [51, 54] Religious group / [54]
Burnout	Age [21] / [36, 49, 54] Gender [21, 54] / [36, 49, 55, 57] Ethnicity / [36, 49, 54, 57] Education / [49, 54] Marital status [36, 57] Income / [49] Religious group / [54]
Stress	Age [42] / [46, 59] Gender / [42, 46, 59] Ethnicity / [59] Education / [42, 59] Marital status [42] / [59]
Peritraumatic dissociation	Age [64] / [38, 39, 59] Gender / [38, 39, 56, 59] Ethnicity / [59] Education / [39, 59] Marital status / [38, 39, 59]
Fatigue	Age [63] Gender [63] Ethnicity / [63] Education / [63]
Quality of life	Age / [63] Gender / [63] Ethnicity / [63] Education / [63]
Hostility	Age / [51, 52] Gender / [52] Ethnicity / [51] Education / [51, 52] Marital status / [51]
Alcohol disorder	Age / [43] Gender / [43] Marital status / [43]
Resilience	Age / [49] Gender / [49] Ethnicity / [49] Education / [49] Income / [49]

**Table 2:** History of mental illness as a predictor of psychological distress following disaster relief work

Disorder	Papers reporting significant association with disorder / papers reporting non-significant association with disorder
PTSD	[27, 29, 30, 32, 34, 35, 45, 65–67] / [68]
Anxiety	[65, 67] / [68]
Depression	[65, 67] / [68]
Psychological distress / morbidity	[65]
Alcohol and drug disorder	[67]

**Table 3:** History of trauma as a predictor of psychological distress following disaster relief work

Disorder	Papers reporting significant association with disorder / papers reporting non-significant association with disorder
PTSD	[27, 29, 35, 47, 49, 54, 62, 65, 66] / [33, 36, 37, 40, 45, 46, 50, 57] Similar / [45, 62, 69] Dissimilar [69] History of life stressors [28, 30, 32, 36, 45] / [29]
Anxiety	[47, 54] / [65]
Depression	[47, 54, 65] / [50, 59]
Psychological distress / morbidity	[62]/ [49, 65] Similar / [69] Dissimilar [69] History of life stressors [60–62]
Stress	[59] / [46]
Somatic symptoms	[47] / [54]
Burnout	[36, 54] / [49, 57] History of life stressors [36]
Peritraumatic dissociation	/ [59]
Resilience	[49]

**Table 4:** Previous experience as a disaster relief worker as a predictor of psychological distress following disaster relief work

Disorder	Papers reporting significant association with disorder / papers reporting non-significant association with disorder
PTSD	[7, 26, 34, 37–39, 42, 70–73] / [29, 39, 48, 50, 51, 65, 68, 74] Success in prior disaster relief work / [65]
Anxiety	/ [48, 51, 65, 68, 74] Success in prior disaster relief work / [65]
Depression	/ [39, 42, 48, 50, 51, 65, 68, 74] Success in prior disaster relief work [65]
Psychological distress / morbidity	[7, 26, 60, 61, 73, 75] / [65, 70] Success in prior disaster relief work / [65]
Stress	/ [42]
Peritraumatic dissociation	[38, 64] / [38, 39]
Somatic symptoms	/ [51]
Hostility	/ [51]

**Table 5:** Personality traits as predictors of psychological distress following disaster relief work

Disorder	Papers reporting significant association with disorder / papers reporting non-significant association with disorder
PTSD	Hardiness [76] Tough mindedness / [68] Extraversion [44] / [45, 68] Neuroticism [32, 44, 45] / [68] Dissimulation / [68] Adjustment [73, 75] Avoidance [45] Psychoticism [44]
Anxiety	Tough mindedness / [68] Extraversion / [68] Neuroticism [68] Dissimulation / [68]
Depression	Tough mindedness / [68] Extraversion / [68] Neuroticism [68] Dissimulation / [68]
Psychological distress / morbidity	Hardiness [61, 76] Conscientiousness [60] Neuroticism [60, 77] Extroversion [60] Adjustment [73, 75]
Peritraumatic dissociation	Adjustment [64] Prudence [64] Ambition [64] Identity [64] Adaptive [64]

**Table 6:** Coping strategies as a predictor of psychological distress following disaster relief work

Disorder	Papers reporting significant association with disorder / papers reporting non-significant association with disorder
PTSD	Confrontive coping / [7, 26] Seeking social support / [7, 26] Self-control / [7, 26] Accepting responsibility / [7, 26] Avoidance [7] / [14] Planned problem solving / [7, 26] Positive reappraisal [7] / [26]
Psychological distress / morbidity	Confrontive coping [7, 26] Seeking social support [14] / [7] Self-control / [7, 26] Accepting responsibility [26] / [7] Avoidance [26] / [7] Planned problem solving [26] / [7] Positive reappraisal [26] / [7]

**Table 7:** Other predictors of psychological distress following disaster relief work

Disorder	Papers reporting significant association with disorder / papers reporting non-significant association with disorder
PTSD	Having children [54, 78] / [36] Locus of control [73, 75] Voluntary status [8, 70, 79] / [58] Professional status [65] / [8, 58, 79] Attachment style [49, 55, 78] Childhood environment [53]
Anxiety	Having children / [54] Voluntary status [79] Professional status / [65, 79]
Depression	Having children [54] / [59] Professional status / [65]
Psychological distress / morbidity	Locus of control [73, 75, 80] Professional status [70] / [58, 65, 79] Voluntary status [79] / [58, 70] Reassured by physicians [63] Attachment style [77, 78] / [49]
Stress	Having children / [59]
Somatic symptoms	Having children / [54]
Burnout	Having children / [36, 54]
Peritraumatic dissociation	Locus of control [64] Having children / [59]
Fatigue	Reassured by physicians [63]
Quality of life	Reassured by physicians [63]
Resilience	Attachment style [49]

## Results

### Study characteristics

The initial search yielded 8386 studies. Thorough reference checking and tracking, including forward citation tracking of each included study and relevant systematic reviews, identified another 40 citations. Sixty-two studies met our inclusion criteria.

The details of each study and their findings are given in Table 1. The majority (*n* = 60) were cross-sectional; just two were prospective. Most (*n* = 41) focussed on large-scale disasters, natural or accidental (e.g. earthquakes, air crashes), 19 studies focussed on terrorism and 14 examined the 11th September attacks in the USA. The included studies used a variety of measures to assess the mental health of all participants, for a brief description of each measure, please see the Main Results Table, Additional file 4.

Overall, 41 different potential predictors were identified. We categorised these as relating to demographics; history of mental illness; history of trauma; previous experience as a disaster relief worker; personality traits; coping strategies; other. The proportion of studies supporting or opposing that a predictor is associated with mental-ill health is reported. Where only one study examined a predictor, it has not been reported in text. Please see the corresponding tables for this data.

### Risk of bias

Most included studies in the review obtained high quality scores (mean: 84.5%; range 68.8 to 100%). Please see the Main Results Table (Additional file 4) for the quality obtained by each study. The most common weaknesses were, not stating the inclusion or exclusion criteria, not describing reasons for loss to follow-up and, not reporting appropriate caveats in the interpretation of results. Given the high quality of the research in this area, it was not possible to weigh the findings of individual studies by quality in our narrative synthesis.

### Associations between demographics and distress and morbidity

Table 1 shows the association between demographics and distress or disorder. There is mixed evidence for an association for age. Most findings revealed no association between age and PTSD (19 out of 34), anxiety (four out of six), depression (nine out of 12), psychological distress/morbidity (six out of 10), somatic symptoms (four out of four), burnout (three out of four, with the latter being a lower quality study [21]), stress (two out of three), peritraumatic dissociation (three out of four) or hostility (two out of two). Still, some studies did identify that being older was associated with stronger severity and higher probability of PTSD (seven out of 34) alongside a worse trajectory of this disorder (two out of 34) and, higher probability of general psychological distress/morbidity (three out of 10). Other studies identified that youth was associated with a higher probability of PTSD (six out of 34), anxiety (two out of six) and depression (two out of 12).

There is mixed evidence for an association for gender. The majority revealed no association between gender and PTSD (17 out of 28), depression (seven out of 10), psychological distress/morbidity (five out of six), peritraumatic dissociation (four out of four), burnout (four out of six, with one of the two contradictory papers being a lower quality paper [21]) and, stress (three out of three). A minority of studies found that being a woman was associated with a higher probability of depression (two out of 10), PTSD (eight out of 28) with a worse trajectory (two out of 28) and, higher severity of burnout (two out of six). Mixed findings were found regarding anxiety and gender; two studies out of four found that being a woman was associated with a higher probability of this condition whilst two others found no association. It is worth noting that for the latter, one of these two studies [21] was one of the few poorer quality studies.

There is mixed evidence for an association for ethnicity. Most findings revealed no association between ethnicity and PTSD (eight out of 13), anxiety (two out of three), general psychological distress or morbidity (three out of three) or burnout (four out of four). However, a minority of papers found that non-White participants were more likely to report PTSD (three out of 13) with a worse trajectory [29, 30]. Mixed findings were found regarding depression and ethnicity; two studies out of four found that non-White participants were associated with a higher probability of this condition whilst two others found no association.

There is little evidence for an association for education. Most findings revealed no association between the level of education and PTSD (13 out of 19), anxiety (two out of three), depression (five out of seven), psychological distress/morbidity (three out of three), somatic symptoms (two out of two), stress (two out of two), peritraumatic dissociation (two out of two), burnout (two out of two), or hostility (two out of two). Still, some studies did find that having less education was associated with a higher probability of PTSD (four out of 19).

There is limited evidence for an association for marital status. Most findings revealed no association between marital status and PTSD (14 out of 22), anxiety (two out of three), depression (four out of seven), psychological distress or morbidity (three out of four), somatic symptoms (two out of three), or peritraumatic dissociation (three out of three). Still, there was some limited evidence that being unmarried, or not cohabiting, was associated with a higher probability of burnout (two out of two) and a higher probability and severity of depression (three out of seven) and PTSD (three out of three) with a worse trajectory [22, 28–30].

There is little evidence for associations between income, social class, or religious affiliation and psychiatric distress or morbidity. The majority of studies found that income was not associated with PTSD (four out of six) nor was it associated with psychological distress, (two out of two).

### Associations between history of mental illness and distress and morbidity

Table 2 shows there to be strong evidence for an association between history of mental illness and distress or disorder. A history of mental illness was associated with higher severity and probability of PTSD (10 out of 11) with a worse trajectory [29, 30, 45], anxiety and depression (for both two out of three).

### Associations between history of trauma exposure and distress and morbidity

Table 3 shows there to be evidence for an association between history of trauma exposure and distress or disorder. Studies found that history of trauma was marginally more associated with higher severity and probability of PTSD (nine out of 17), anxiety (two out of three), and depression (three out of five); the evidence was mixed for burnout (two our of four). A history of trauma that was similar to the trauma experienced during the disaster relief work was not associated with PTSD (three out of three). There is strong evidence that a history of life stressors is a predictor for psychiatric distress or disorder post-deployment. Studies described life stressors as, for example, “death of, or separation from, close relatives, major systemic disease or problems at work” [28, 60]. History of life stressors was associated with higher probability of PTSD (nine out 17) and psychological distress and morbidity (three out of three).

### Associations between previous experience with disaster relief work and distress and morbidity

Table 4 shows there to be mixed evidence for an association between previous experience with disaster relief work and psychiatric distress or disorder. The majority of studies found that less experience was associated with higher post-trauma rates of PTSD (nine out of 11), psychological distress (two out of six) and peritraumatic dissociation (two out of four). Other studies found that more experience was associated with higher rates of PTSD (three out of 11) and general psychological distress and morbidity (four out of six). Other studies found no association between experience and PTSD (eight out of 19), anxiety (five out of five), depression (eight out of eight) or psychological distress or morbidity (two out of two).

### Associations between personality traits and distress and morbidity

Table 5 shows there to be mixed evidence for an association between personality traits and psychiatric distress or disorder. Hardiness, defined as believing one is in control of one’s life, that commitment to goals results in positive outcomes, and that daily stressors should be viewed as challenges [76], was associated with lower levels of psychological distress (two out of two). Neuroticism was associated with a higher probability of PTSD (three out of four with the fourth [68] being of lower quality rating), and severity of psychological distress (two out of two). Two studies, one [68] of lower quality rating, found that extraversion was not associated with PTSD (two out of three), but that adjustment, defined as emotional stability [73], was associated with PTSD, though not with psychological distress/morbidity (two out of two).

### Associations between coping mechanisms and distress and morbidity

Table 6 shows the association between coping mechanisms with disaster relief work and distress or disorder. Fifteen different components of coping were tested by only two studies, with all findings revealing no or conflicting evidence.

### Associations between other factors and distress and morbidity

Table 7 shows there to be mixed evidence for an association between other factors and distress or disorder. Having children was not associated with burnout (two out of two). Having an external locus of control, defined as believing outside forces are in control, was associated with higher rates of PTSD (two out of two) and psychological distress (three out of three). Voluntary status when engaging with disaster relief work was associated with higher PTSD rates (three out of four), but not psychological distress (two out of three) and, professional status was not associated with PTSD (three out of four) anxiety (two out of two), or psychological distress (three out of four).

## Discussion

To the best of our knowledge, this is the first systematic review investigating the potential usefulness of pre-employment and pre-deployment screening for predictors of psychological distress or disorder amongst disaster relief staff. We were able to identify and combine the findings from 62 studies. In general, demographics, previous experience as a disaster relief worker, personality traits, coping strategies, history of trauma, and having children have little evidence to support them as predictors of psychological vulnerability. Conversely, voluntary status, previous history of mental illness and previous life stressors spanning from in the past 3 months to throughout life, did emerge as reliable predictors. Locus of control, attachment style, childhood environment, and whether a person is reassured by physicians when unwell and experiencing symptoms were also identified as possible predictors and might be worthy of additional research as relatively few studies have assessed these features.

Our findings that gender, ethnicity, education and marital status, were not associated with psychological vulnerability was surprising. These results deviate from previous findings. Both being a woman and belonging to an ethnic minority have often been identified as vulnerability factors for psychopathology as such individuals may be more susceptible to life-time stress [79, 82]. Similarly, our finding that those with lower education were not more at risk of psychological distress is contrary to other researcher’s views that less educated people are more vulnerable to trauma because they have a reduced capacity to make sense of difficult experiences [83]. It was also unexpected to find that marital status was not associated with vulnerability; being married or in a long term relationship has been assumed to be associated with greater social support, which in turn is associated with reduced vulnerability to distress [84]. These findings suggest that such demographic variables are less relevant as predictors of distress or disorder among those people who choose to work in the field of disaster response, perhaps reflecting a generally high level of resilience among the group.

Our findings reveal clear evidence that individuals with a history of mental illness are more vulnerable to psychological distress following disaster relief work. Individuals who have a history of trauma and/or significant life stressors are also susceptible, although the evidence on this was less clear cut. These findings do not correspond with those of a systematic review exploring emergency service workers [15], which found that workers with pre-existing mental illnesses or trauma were no more vulnerable than those without. This suggests that emergency services have procedures in place, such as mental health training for managers [85], to protect employees with such predisposing factors. It is likely that such procedures are not present for disaster relief workers.

Alternatively, the divergent findings might be explained by differences in the recruitment procedures for the two roles, resulting in qualitatively different populations, differences in training, or differences in the type of stressors the two groups are exposed to. Practical difficulties exist in basing pre-deployment screening on these findings however. First, those studies that explored life stressors used a range of time-periods in their measures, from life-stressors experienced in the past three to 6 months to those experienced at any point in the participant’s life. Excluding or supporting all employees who have ever experienced a life stressor would be impractical and probably detrimental; it would prevent people who have experienced prior challenges from using their experience as a disaster worker. The evidence from this review suggests that people who have experienced significant life stressors should have their fitness to deploy carefully scrutinised and their mental health carefully monitored. Second, although a robust association was found between a history of mental illness and future mental health problems following humanitarian or disaster work, it remains unclear whether the increased risk of developing mental health problems post deployment is only related to recent episodes of ill mental health or not. Once again, we suggest that having a prior mental health history should not be seen as a bar to deployment, but as an indicator that someone’s fitness to deploy should be carefully scrutinised by suitably experienced occupational health professionals and their mental health carefully monitored.

As suggested by Brooks and colleagues [86], volunteers in our review were found to be more prone to post-disaster psychopathology than those with a professional status. This may be linked to their lack of experience or training or that the organisations that make use of volunteer workers do not have well-established support mechanisms in place compared to those who employ professional disaster responders. Our results suggest that organisations should take particular care to prepare, support and monitor all disaster response staff, volunteer or professional. Methods for enabling this, such as organisational training and mental health education are discussed below.

Further research is needed before utilising many other potential predictors identified in this review for identifying pre-deployment vulnerability. These include parental status, attachment styles, childhood environment, external locus of control, and being reassured by physicians, all of which were found to increase vulnerability, although only in a small number of studies.

Few studies in this review investigated the impact of social support on psychological distress, however, the findings do suggest that it is an effective protective factor amongst relief workers [7, 26]. This is supported by the literature; perceived and actual social support has the potential to alleviate psychological symptoms [87–90]. Indeed, it is vital that relief work organisations create a culture of social support and openness about psychological difficulties amongst rescue workers [88, 89].

One approach is to encourage organisations to develop policy on mental health education for relief workers pre- deployment. Our results suggest that such education should focus on mental health literacy, the risk relief work poses, possible risk factors (e.g. prior life stressors and/or prior mental health problems), early warning signs, relaxation techniques and, available support services and their contact details; such education would enable recognition of ill mental health and swift action-taking [88]. Organisations would be well advised to encourage staff to talk about mental health which may help empower them to seek support [88, 89]. We would also suggest ensuing that program managers have access to mental-health training and well-developed protocols for differential responses based on the level of presenting problems and any identified risk to self or others [85]. As a result of these efforts, volunteers and professionals would feel well-prepared and supported and the culture of openness and mental health literacy further enforced [88].

Overall, our findings suggest that, whilst employing organisations might consider it appealing to screen for resilience among staff who wish to engage in crisis response or disaster relief work, this may well not be an effective way to protect the mental health of disaster relief teams. With few exceptions, the evidence base underlying the variables that might be included in such screening is weak. Given that risks also exist for screening, including the risk of inappropriately excluding skilled staff and of enhancing stigma or discrimination against those identified as high risk [86], employers may wish to exert caution when using screening tools until better quality evidence relating to their use become available. Our findings suggest that in some cases, careful scrutiny of individuals who appear vulnerable by appropriately skilled occupational health staff is warranted.

### Strengths and limitations

The main strengths of this review are the detailed systematic search strategy, which identified multiple relevant studies, and the high quality of the studies included in the review.

As with all systematic reviews, however, there is a risk of publication bias and selective reporting. The risk of publication bias was heightened by our decision to only include studies published in peer-reviewed journals and published in English. The exclusion of foreign-language or grey literature reports may have altered our conclusions.

Synthesis was made challenging in our review by the vague terminology used in some papers, the use of multiple outcome measurement tools, and the range of techniques used to define an adverse outcome. In particular, the use of distress as the primary endpoint in many studies posed a difficulty. Distress, at least in the short-term, is a normal response to a traumatic exposure and does not necessarily indicate the presence of any mental health disorder. On this basis, pre-employment screening for risk factors for distress may be uncalled for. On the other hand, distressed employees may require additional support from their colleagues and line manager, making identification of risk factors a worthwhile exercise. In this review, we elected to include studies relating to both distress and disorder. Although we include both types of outcome in our synthesis, our impression is that excluding distress would not have dramatically altered our conclusions. It would be helpful if future studies could look at the risk of experiencing longer term illness rather than short term distress.

The majority of the studies included in this review were cross-sectional, relying on retrospective recall of pre-employment and pre-deployment predictors thus leaving the studies vulnerable to recall bias. More prospective studies are required if further investigation into risk factors is desired.

The common use of self-report measures to assess post-deployment symptomatology also leaves studies open to criticism, as this is not equivalent to diagnosis by a qualified clinician. However, a recent meta-analysis of PTSD prevalence amongst emergency service workers found that both self-report and diagnostic interviews provided similar results [91].

## Conclusion

Identifying members of staff who are likely to experience distress or disorder following disaster relief work is a laudable aim. However, in practice, the current evidence base does not support the use of pre-employment and pre-deployment screening as a method of protecting disaster workers’ mental health. Our results do support disaster response organisations ensuring that volunteers are as well prepared and supported as professional disaster works. Additionally, people with a prior history of mental illness, or who have recently experienced significant life stressors, should be scrutinised by occupational health staff to ensure that any associated risks are properly managed. However, our results suggest that until better evidence becomes available, there is no role for the use of pre-employment and pre-deployment mental health screening for this population.

## Supplementary information

**Additional file 1.** Combined search terms

**Additional file 2.** Quality appraisal tool for quantitative studies

**Additional file 3.** PRISMA 2009 Flow Diagram

**Additional file 4.** Main Results Table.

## Data Availability

The datasets supporting the conclusions of this article are included within the article and its additional files.

## Abbreviations

PTSD: Post-traumatic stress disorder
USA: United States of America
EO: Elena Opie
SKB: Samantha Brooks
NG: Neil Greenberg
GJR: G. James Rubin
