# Supplementary material for: The usefulness of pre-employment and pre-deployment psychological screening for disaster relief workers: a systematic review
Source: BMC Psychiatry. 2020 May 11;20:211. doi: 10.1186/s12888-020-02593-1 (PMC7216600; doi:10.1186/s12888-020-02593-1)
Supplement: Supplementary file 1 — Additional file 1. Combined search terms [file 12888_2020_2593_MOESM1_ESM.docx]

Additional file 1. Combined search terms

| **Database** | **Emergency personnel AND** | **Pre-deployment screening AND** | **Outcome AND** | **Cohort study** |
| --- | --- | --- | --- | --- |
| PsycINFO;  MEDLINE;  EMBASE;  GlobalHealth | Deployment | ‘Psychological assessment*’ | PTSD | Cohort |
|  | Humanitarian | ‘Psychological evaluation*’ | Post-trauma* | Longitudinal |
|  | Military | ‘Psychological test*’ | Post trauma* | Follow up |
|  | Army | ‘Psychological screen*’ | Posttrauma* | Followup |
|  | Relief work | ‘Psychological exam*’ | Trauma | Prospective |
|  | Reliefwork | ‘Psychological measure*’ | Trauma* | Retrospective |
|  | Relief work | ‘Psychological tool*’ | Psychotrauma* |  |
|  | Reliefwork | ‘Psychiatric assessment*’ | War |  |
|  | Disaster respons* | ‘Psychiatric evaluation*’ | Combat |  |
|  | Emergency medical service* | ‘Psychiatric test*’ | Traumatic stress |  |
|  | Emergency medical | “Psychiatric screen*” | Neuros* |  |
|  | Emergencymedical | “Psychiatric Exam*” | Neurotic |  |
|  | Emergency respons* | ‘Psychiatric Measure*’ | Depress* |  |
|  | Emergency respond* | ‘Psychiatric tool*’ | Depressivedisorder* |  |
|  | Emergencyrespond* | ‘Mental health assessment*’ | Panic |  |
|  | Emergency personnel | ‘Mental health evaluation*’ | Panicdisorder* |  |
|  | Emergencypersonnel | ‘Mental health test*’ | Anxiety |  |
|  | Emergency worker* | ‘Mental health screen*’ | Anxietydisorder* |  |
|  | Emergencyworker* | ‘Mental health exam*’ | Stress* |  |
|  | Emergency service* | ‘Mental health measure*’ | Stressdisorder* |  |
|  | Emergencyservice* | ‘Mental health tool*’ | Shell shock |  |
|  | First responder* | Personnel select* | Shellshock |  |
|  | Firstresponder* | Personnelselect* | Psychiatric |  |
|  | Personnelrecruit* | Personnel recruit* | Mental health |  |
|  | Paramedic* | Personnelrecruit* | Mentalhealth |  |
|  | Disaster respond* | Mentalhealth | Mental illness |  |
|  | Disasterrespond* | Mental illness | Mentalillness |  |
|  |  | Risk factor* | Wellbeing |  |
|  |  | Riskfactor* | Well being |  |
|  |  | Predict* | Mental disorder* |  |
|  |  | Personality | Mood disorder* |  |
|  |  |  | Affective disorder* |  |
|  |  |  | Affective symptom* |  |
|  |  |  | Emotional disorder* |  |
|  |  |  | Adjustment disorder* |  |
|  |  |  | Psycholog* |  |
