# Supplementary material for: The usefulness of pre-employment and pre-deployment psychological screening for disaster relief workers: a systematic review
Source: BMC Psychiatry. 2020 May 11;20:211. doi: 10.1186/s12888-020-02593-1 (PMC7216600; doi:10.1186/s12888-020-02593-1)
Supplement: Supplementary file 3 — Additional file 3. PRISMA 2009 Flow Diagram [file 12888_2020_2593_MOESM3_ESM.docx]

Additional file 3. PRISMA 2009 Flow Diagram


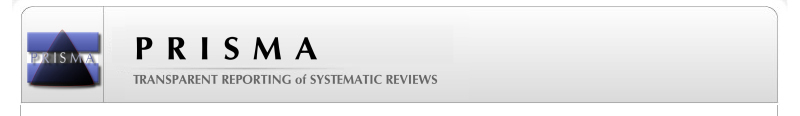


Studies included in quantitative synthesis
(n = 62)

Full-text articles assessed for eligibility
(n = 227)

Records after duplicates removed
(n = 5,627)

## Included

## Eligibility

## Screening

## Identification

Records identified through database searching
(n = 8,386)

Additional records identified through reference checking and tracking
(n = 40)

Records excluded
(n = 5,407)

- Any studies that did not correspond to the specified inclusion criteria were excluded

Titles and abstracts of records screened
(n = 5,627)

Full-text articles excluded
(n = 165)

- Studies based on military samples (combat and non-combat) (n=149)
- Not based on a specific disaster (n=4)
- Did not report on pre-deployment factors (n=7)
- Did not use a validated measure (n=3)
- Did not assess for a psychological disorder (n=2)
