# Supplementary material for: The usefulness of pre-employment and pre-deployment psychological screening for disaster relief workers: a systematic review
Source: BMC Psychiatry. 2020 May 11;20:211. doi: 10.1186/s12888-020-02593-1 (PMC7216600; doi:10.1186/s12888-020-02593-1)
Supplement: Supplementary file 4 — Additional file 4. Main Results Table. [file 12888_2020_2593_MOESM4_ESM.docx]

Additional file 4. Main Results Table.

| **Author, year of publication; disaster description; country of study** | **Study design; time assessed since deployment/follow-up time** | **Population sample**  Sample total (*n*)  Gender split (w:m)  Age range (years)  Mean age (years)   Job role (*n*) | **Disorder** **outcome** (*measure used*) | **Predictive factors** (*measure*)  **Bold** = significant association | Quality score (%) |
| --- | --- | --- | --- | --- | --- |
| Adams et al., 2008  World Trade Centre  United States  (22) | Cross-sectional  20months | *n*=236  189:47  Undisclosed  >50  Social workers | Secondary trauma (CFS)  Burnout (CFS) | Age  Gender  Ethnicity  **Marital status**: not married associated with higher rates of burnout  **History of trauma**: less associated with higher rates of burnout  **History of life stressors**: less associated with higher rates of secondary trauma and burnout  Having children | 86.67% |
| Alexander & Wells, 1991  Piper Alpha North Sea oil-rig disaster  United Kingdom  (69) | Prospective  Undisclosed time pre-deployment to 3months post | *n*=48  8:63  Undisclosed  Undisclosed  Police: body handling on site, at mortuary, or both | PTSD (IES)  Anxiety and depression (HAD) | History of mental illness (HAD)  Previous experience as a disaster relief worker  **Personality traits** (EPI): neuroticism associated with higher rates of anxiety and depression | 68.80% |
| Alvarez & Hunt, 2005  World Trade Centre  United States  (65) | Cross-sectional  6months  Samples included deployed (*n*=82); control non-deployed (*n*=32) | *n*=82 deployed handlers *n*=32 control group  65:49 (control group included)  Undisclosed  43 (control group included)  Canine search and relief handlers | PTSD (*PSS-SR; PSS-I*)  Anxiety (*BAI*)  Depression (*BDI*)  Psychological distress (*BSI*) | **History of mental illness** (*SCID-I/P*): associated with higher rates of PTSD, anxiety, depression, psychological distress  **History of** **trauma** (*SCID-I/P*): associated with higher rates of PTSD and depression  Previous experience and **success** as a canine search and relief handler (*interview*): associated with lower rates of depression  **Emergency-work certification**: associated with lower rates of PTSD | 86.67% |
| Armagan et al., 2006  Tsunami  Turkey  (23) | Cross-sectional  1month | *n*=33  16:17  Undisclosed  30  Turkish Red Crescent disaster relief | PTSD (*CAPS-1*) | Age  **Gender**: women associated with higher rates of PTSD  History of trauma  **Experience of disaster relief work**: less associated with higher rates of PTSD | 93.33% |
| Bartone et al., 1989  Gander aviation disaster  United States  (78) | Cross-sectional, 2 waves  6months, 1year | *n*=131  9:122  22 to 51  34  Victim’s family assistance workers | PTSD (*HSC*)  Psychological distress (*StS; BS*) | **Personality traits** (hardiness) (*KPH-M*): associated with lower rates of PTSD and psychological distress | 86.67% |
| Ben-Ezra et al., 2008  Bet-Yohshua train crash  Israel  (25) | Cross-sectional  24hours | *n*=23  13:10  20.3  19-23  Relief personnel | PTSD (*IES-R*)  Depression (*CES-D*)  Peritraumatic dissociation (*DES*) | Age  Gender  Education  Marital status  Experience as a disaster relief worker | 86.67% |
| Ben-Ezra et al., 2006  Gas pipe explosion  Israel  (60) | Cross-sectional  36-48hours | *n*=25  16:9  18-20  19.2  Relief personnel | PTSD (*IES*)  Peritraumatic dissociation (*DES*) | Gender | 80% |
| Ben-Ezra et al., 2005  Hilton Hotel bombing  Israel  (24) | Cross-sectional  96hours | *n*=26  2:24  19 to 34  21.9  Relief personnel | PTSD (*IES*)  Peritraumatic dissociation (*DES*) | Age  Gender  Marital status  **Experience as a disaster relief worker**: less associated with higher rates of PTSD and dissociation | 93.33% |
| Biggs et al., 2010  World Trade Centre  United States  (48) | Cross-sectional  2-3weeks | *n*=90  22:68  Undisclosed  35.6  Relief personnel | Depression (*ZSRDS*)  Acute stress disorder (*undisclosed, validated*)  Peritraumatic dissociation (*PDEQ*) | **Age**: younger associated with higher depression morbidity  Gender  **Ethnicity**: non-White associated with higher depression morbidity  Education  Marital status  **History of trauma**: associated with higher acute stress disorder morbidity  Having children | 86.67% |
| Boscarino et al., 2004  World Trade Centre  United States  (61) | Cross-sectional  20months | *n*=236  182:47  <49 to 60+ | Secondary trauma (*CFS-R*)  Burnout (*CFS-R*) | Gender  Ethnicity  Marital status  History of trauma | 80% |
| Bowler et al., 2012  World Trade Centre  United States  (43) | Cross-sectional, 2 waves  2-3years, 5-6years | *n*=2,940  413:2,527 | PTSD (*PCL*)  Psychological distress (*KPDS; PDI*) | **Age**: older associated with higher PTSD morbidity  **Gender**: women associated with higher PTSD morbidity and higher rates of psychological distress  Ethnicity  Income | 86.67% |
| Bowler et al., 2010  World Trade Centre  United States  (49) | Cross-sectional  2-3years | *n*=4,017  582:3,435  18 to 65+  25 to 44  Police working as disaster workers | PTSD (*PCL*) | **Age**: for both sexes, older associated with higher PTSD morbidity  **Gender**: women associated with higher PTSD morbidity  **Ethnicity**: for men, Hispanic, Asian or multi-racial ethnicities, compared to White ethnicity, associated with higher PTSD morbidity  **Education**: for women, < college degree associated with higher PTSD morbidity | 100% |
| Brown et al., 2002  Political violence  Northern Ireland  (80) | Cross-sectional  During event | *n*=300  62:248  35.82  18 to 60  Firefighters | Psychological distress (*GHQ*) | **Locus of control** (*LOC*): external locus of control associated with higher rates of psychological distress | 80% |
| Chang et al., 2008  Chi-Chi earthquake  Taiwan  (50) | Cross-sectional  2-5months | *n*=193  0:193  22 to 57  33.7  Firefighters as disaster workers and body handlers | PTSD (*IES*)  Psychological morbidity (*CHQ*) | **Age**: being older associated with higher PTSD and psychological morbidity  Marital status  **Experience as a disaster relief worker**: >3 years associated with higher rates of PTSD and higher psychological morbidity  **Coping style** (*WCQ*): confrontive coping, distancing, seeking social support, accepting responsibility, escape-avoidance, planned problem solving, positive reappraisal associated with lower psychological morbidity | 86.67% |
| Chang et al., 2003  Chi-Chi earthquake  Taiwan  (7) | Cross-sectional  5months | *n*=84  0:84  20 to 56  27.6 ± 7.9  Firefighters working as disaster workers in relief and body handling | PTSD (*IES*)  Psychological morbidity (*CHQ*) | **Age**: older associated with higher rates of PTSD and psychological morbidity  **Marital status**: married associated with higher psychiatric morbidity  **Experience as a disaster relief worker**: >3 years associated with higher rates of PTSD and higher psychological morbidity  **Coping style** (*WCQ*): distancing, escape-avoidance associated with higher rates of PTSD and positive reappraisal associated with lower rates of PTSD; confrontive coping associated with higher psychological morbidity | 93.33% |
| Creamer & Liddle, 2005  World Trade Centre  United States  (26) | Cross-sectional  3-5months | *n*=80  50:30  27 to 77  50  Disaster mental health workers | Secondary traumatic stress (*IES*) | Age  Gender  Education  Marital status  History of trauma (*LEC*) | 86.67% |
| Cukor et al., 2011  World Trade Centre  United States  (66) | Cross-sectional  10-34months | *n*=2,960  92:2868  Undisclosed  45.2  Utility workers working as non-relief disaster relief and recovery workers | PTSD (*CAPS; PCL*; *SCID; BSI; BDI-II*) | **Age**: associated with PTSD morbidity, direction undisclosed  **Gender**: associated with PTSD morbidity, direction undisclosed  **History of mental illness**: associated with higher PTSD morbidity  **History of trauma**: associated with higher PTSD morbidity | 80% |
| Dobashi et al., 2014  Earthquake  Japan  (27) | Cross-sectional  1month | *n*=605  6:599  19 to 50+  19-29  Japan ground self-defence force | PTSD (*IES-R*)  Psychological distress/morbidity (*KPDS*) | **Age**: younger associated with higher psychological morbidity  Gender | 93.33% |
| Dougall et al., 2000  Aviation disaster  United States  (70) | Cross-sectional, 4 waves  1-2, 6, 9, 12months | *n*=108  38:70  20 to 68  36  Volunteer healthcare workers; emergency medical technicians; radiologists; dental students; firefighters; USAir maintenance workers, flight attendants, administrative personnel | PTSD (*ITQ*)  Psychological distress (*GSI*) | **History of trauma;** similar and **dissimilar** (*THQ*): associated with higher rates of PTSD and psychological distress | 93.33% |
| Dyregrov et al.,1996  Tour bus crash  Norway  (71) | Cross-sectional, 2 waves  1 to 13months | *n*=43  Voluntary: 3:21 Professional: 7:25  Voluntary: 24 to 56 Professional: 23 to 61  Voluntary: 37.3 Professional: 37.1  Voluntary helpers (*n*=24): Red Cross workers, voluntary fire brigade Professional helpers (*n*=32): police, fire relief personnel, health personnel | PTSD (*IES*)  Psychological distress (*GHQ*) | **Experience as a disaster relief worker**: voluntary status with little experience associated with higher rates of PTSD-avoidance; professional status with much experience associated with lower rates of PTSD-avoidance  **Voluntary or professional status**: at 1month, voluntary status associated with higher rates of PTSD; at 13months, voluntary status associated with higher PTSD-avoidance rates; at 13months, professional status associated with higher rates of psychological distress | 73.33% |
| Ehring et al., 2011  Major earthquake  Northern Pakistan  (40) | Cross-sectional  24months | *n*=267  43:224  Undisclosed  28.93  Social organisers (n= 133); engineers and reconstruction workers (n= 80); coordinators and supervisors (*n*=46) | PTSD (*IES-R*)  Depression and anxiety (*PADQ*)  Somatic symptoms (*BSI^1^*)  Burnout (*MBI*) | **Age**: younger associated with higher depression and anxiety morbidity  **Gender**: women associated with higher PTSD morbidity, anxiety, depression, and higher rates of somatic symptoms and burnout  Ethnicity  Education  Religious group  **History of trauma** (*PTS*): associated with higher PTSD morbidity, anxiety, depression, rates of burnout  **Having children**: with children was associated with lower PTSD and depression morbidity | 86.67% |
| Epstein et al., 1998  Ramstein air disaster  Germany  (56) | Cross-sectional, 3 waves  6, 12, 18months | *n*=355  125:229  19 to 59  31.9  Military medical health care workers | PTSD (*SC-90-R; IES*) | **Age**: at 6, 12, 18months, being younger was associated with higher PTSD morbidity  Gender  Ethnicity  **Education**: at 6, 12, 18months, < bachelor’s degree associated with higher PTSD morbidity | 80% |
| Ersland et al., 1989  “Alexander L. Kielland” oil rig disaster  Norway  (72) | Cross-sectional  9months | *n*=134  Undisclosed  21 to 54  33  Professional disaster workers (*n*=24) including police (*n*=12), aircraft crew (*n*=8), vessel crew members (*n*=2), divers (*n*=1), unclassified (*n*=1);  Non-professional disaster workers (*n*=101) including rig-workers (*n*=33), vessel crew (*n*=31), divers (*n*=13), nurses (*n*=10), catering (*n*=6), physicians (*n*=5), pilots (*n*=2), other (*n*=1); unclassified (*n*=9) | PTSD (*IES*) | **Experience as a disaster relief worker**: having never worked, or having worked few times only, associated with higher rates of PTSD | 80% |
| Evans et al., 2009  World Trade Centre  United States  (67) | Cross-sectional  17-27months | *n*=842  41:801  Undisclosed  45.4  Disaster relief workers | PTSD (*CAPS*) | **History of mental illness** (*SCI*): associated with higher PTSD morbidity  **History of trauma** (*THQ*): associated with higher PTSD morbidity | 73.33% |
| Feder et al., 2016  World Trade Centre  United States  (53) | Cross-sectional, 4 waves  3, 6, 8, 12months | *n*=4,487  Police: 274:1600; Non-responders: 357:2256  Undisclosed Police: 41.7;  non-traditional responders: 46.2  Police (*n*=1874);  Non-traditional responders (*n*=2613) | PTSD (*PCL-S*) | *Police : non-traditional responders*  **Age**: older associated with higher chronic PTSD morbidity  **Gender**: women associated with higher worsening and chronic PTSD morbidity  **Ethnicity**: Hispanic associated with higher improving and chronic PTSD morbidity; ‘other’ associated with higher worsening PTSD morbidity : Hispanic associated with higher improving, worsening, steeply worsening and chronic PTSD morbidity; ‘other’ associated with steeply worsening PTSD morbidity  **Education**: : ≤ high school associated with higher improving, worsening, steeply worsening and chronic PTSD morbidity  **Marital status**: : widowed, separated or divorced associated with higher chronic PTSD morbidity  Income  **History of mental illness** (depression, anxiety, or PTSD): associated with higher improving and worsening rates of PTSD morbidity : associated with higher improving, worsening, steeply worsening and chronic PTSD morbidity  **History of trauma**: associated with higher worsening and chronic PTSD morbidity  History (<1year) of life stressors  Experience as a disaster relief worker | 93.33% |
| Fullerton et al., 2004  Aviation disaster  United States  (28) | Cross-sectional, 3 waves  2, 7, 13months | *n*=207  24:183  Undisclosed  36.5  Disaster workers | PTSD (*DSM-IV PTSD Scale*)  Depression (*ZSRDS*)  Acute stress disorder (*undisclosed, validated*) | **Age**: younger associated with higher rates of acute stress disorder  Gender  Education  **Marital status**: unmarried associated with higher rates of acute stress disorder; at 13months, unmarried associated with higher rates of PTSD and depression  **Experience as a disaster relief worker**: at 13months, associated with higher rates of PTSD | 93.33% |
| Grieger et al., 2003  Virginia sniper attacks  United States  (29) | Cross-sectional  2-3weeks | *n*=382  210:172  Undisclosed  39 ± 10.9  Hospital staff | PTSD (*ASDI*)  Depression (*PHQ-9*)  Alcohol disorder (*CAGE*) | Age  **Gender**: women associated with higher rates of PTSD  Marital status  History of substance abuse | 86.67% |
| Guo et al., 2004  Chi Chi earthquake  Taiwan  (8) | Cross-sectional  1month | *n*=252  0:252  Undisclosed  Professional:  37.9 ± 8.7; Non-professional: 22.1 ± 1.2  Professional disaster workers, firefighters (*n*=167); non-professional volunteers, soldiers (*n*=85) | PTSD (*DTS-C; SPAN-C*) | Age  Gender  Education  Marital status  **Voluntary** or professional status: volunteer status associated with higher PTSD-avoidance morbidity | 73.33% |
| Hagh-Shenas et al., 2005  Bam earthquake  Iran  (81) | Cross-sectional  90days | *n*=154  Undisclosed  Undisclosed  Student volunteers: 21.1 Firefighters: 34.2  Red crescent: 22.8  Student volunteers (*n*=100); firefighters (*n*=36); red crescent (*n*=18) | PTSD (*ESHEL*)  Anxiety sensitivity (*ASI*)  Psychological distress (*GHQ-28*) | **Voluntary** or professional status: volunteer status associated with higher PTSD morbidity, higher rates of anxiety sensitivity, psychological distress | 73.33% |
| Hodgkinson & Shepherd, 1994  Piper Alpha North Sea oil-rig disaster; Clapham Rail crash  Britain  (44) | Cross-sectional, 2 waves  4-9, 12months | *n*=39  39:0  26 to 61  40.3  Social workers | Psychological distress (*HSCL*; *PWS*) | Age  Gender  Marital status  **History (<1year) of life stressors**: (*SRRS*): associated with higher rates of psychological distress  **Experience as a disaster relief worker**: more associated with higher rates of psychological distress on the HSCL Anxiety subscale  **Personality traits** (hardiness) (*PHS*): low hardiness associated with higher rates of psychological distress | 73.33% |
| Huang et al., 2013  Wenchuan earthquake  China  (30) | Cross-sectional  18months | *n*=923  0:923  18 to 28  Undisclosed  Military relief workers | PTSD (*CAPS*) | Age  Ethnicity  Education  Marital status  **Personality traits** (*EPQ*): neuroticism and psychoticism associated with higher PTSD morbidity; extraversion associated with lower PTSD morbidity | 86.67% |
| Hytten & Hasle, 1989  Hotel fire relief operation  Norway  (73) | Cross-sectional  3days | *n*=58  0:58  22 to 59  37.8  Non-professional firefighters | PTSD (*IES*) | **Experience as a disaster relief worker**: less associated with higher rates of PTSD | 73.33% |
| Jayasinghe et al., 2008  World Trade Centre  United States  (51) | Cross-sectional  Undisclosed | *n*=1,040  49:981  Undisclosed  45.9  Disaster relief workers | PTSD (*CAPS; PLC*) | **Age**: older associated with higher rates of PTSD  **Marital status**: unmarried associated with higher rates of PTSD  **History of mental illness**: associated with higher rates of PTSD  **History of trauma** (*TEI*): associated with higher rates of PTSD | 73.33% |
| Liao et al., 2002  Major earthquake  Taiwan  (55) | Cross-sectional  2months | *n*= 836  0:836  28.32 ± 8.06  Undisclosed  Disaster workers | Psychological distress (*BSRS; GSI*) | **Age**: older associated with higher rates of psychological distress  **History of life stressors** (<6): associated with higher rates of psychological distress  **Experience as a disaster relief worker**: associated with higher rates of psychological distress  **Personality traits** (*MPI*): moodiness, anxiety-proneness (strongest), conscientiousness, neuroticism associated with higher rates of psychological distress; activity, sociability, extroversion associated with lower rates of psychological distress | 80% |
| Lu et al., 2006  SARS outbreak  Taiwan  (79) | Cross-sectional  During | *n*=135  Undisclosed  Undisclosed  32  Healthcare workers | Psychological morbidity (*C-GHQ*) | **Personality traits** (*EPQ*): neuroticism associated with higher psychological morbidity  **Attachment style** (*PBI*): maternal attachment associated with lower psychological morbidity | 80% |
| Lundin & Bodegard, 1993  Armenian earthquake  Armenia  (64) | Cross-sectional, 3 waves  Immediately, 1month, 9months | *n*=50  Undisclosed  22 to 55  30-49 | PTSD (*IES*)  Psychological distress (*GHQ-9*) | Education  Voluntary or professional status | 73.33% |
| Marmar et al., 1999  Interstate-880 Freeway Collapse  United States  (74) | Cross-sectional 3.5years | *n*=322  34:288  Undisclosed  38.7  Emergency medical personnel, road workers, maintenance, police, firefighters | PTSD (*IES-R; M-PTSD*)  Psychological distress (*SC-90-R GSI; SC-90-R somatization subscale*) | **Experience as a disaster relief worker**: less associated with higher rates of PTSD and psychological distress  **Personality traits** (adjustment, *HPI*): associated with higher rates of PTSD-avoidance and psychological distress  **Locus of control** (*LOC*): external associated with higher rates of PTSD and psychological distress | 93.33% |
| Marmar et al., 1996  Nimitz Freeway Collapse  United States  (76) | Cross-sectional  Undisclosed time | *n*=157  37:321 (control group included)  21 to 64  38.3  Disaster relief workers: firefighters (*n*=61), police (*n*=114), paramedics (*n*=86), transportation workers (*n*=97) | Peritraumatic dissociation (*PDEQ-SV*) | **Age**: younger associated with higher rates of peritraumatic dissociation  **Experience as a disaster relief worker**: less associated with higher rates of peritraumatic dissociation  **Personality traits** (*HPI*): prudence, ambition, adjustment, identity and adaptive traits associated with lower rates of peritraumatic dissociation  **Locus of control** (*LOC*): external associated with higher rates of peritraumatic dissociation | 86.67% |
| Maunder, 2004a  SARS outbreak  Canada  (62) | Cross-sectional  2-3months | *n*=1,557  1,162:395  Undisclosed  40.2  Healthcare workers | PTSD (*IES*) | **Having children**: associated with higher rates of PTSD  **Attachment style** (*ECR-R*): insecure attachment associated with higher rates of PTSD | 80% |
| Maunder et al., 2006  SARS outbreak  Canada  (63) | Cross-sectional  13-26months | *n*=587  505:82  Undisclosed  Undisclosed | PTSD (*IES*)  Psychological distress (*KPDS*)  Burnout (*MBI*) | **Gender**: women associated with higher PTSD morbidity  **Attachment style**: attachment anxiety associated with higher rates of psychological distress and higher PTSD morbidity; attachment avoidance associated with higher rates of psychological distress | 73.33% |
| McFarlane, 1989  Extreme bushfire  Australia  (57) | Cross-sectional, 4 waves  4, 11, 29months | *n*=315  Undisclosed  Undisclosed  36.1  Firefighters | PTSD (*IES; GHQ*) | **Age**: at 11 and 29months, younger associated with higher PTSD morbidity  Social class  **History of mental illness**: at 4 and 29months associated with higher PTSD morbidity  **History of life stressors**: at 4months life events associated with higher PTSD morbidity  **Personality traits** (*EPI*): at 4, 11 and 29months, neuroticism associated with higher PTSD morbidity | 93.33% |
| McFarlane, 1988  Extreme bushfire  Australia  (31) | Cross-sectional 3 waves  4, 11, 29months | *n*=315  Undisclosed  Undisclosed  35.1 ± 10.6  Firefighters | PTSD (*IES; GHQ*) | Age  **History of mental illness**: associated with higher rates of persistent chronic, resolved chronic, recurrent chronic PTSD  History of trauma, similar  **History of life stressors**: associated with higher persistent chronic PTSD morbidity  **Personality traits** (*EPI*): neuroticism associated with higher persistent chronic PTSD; avoidance associated with higher acute, persistent chronic, resolved chronic and, at 11months, delayed-onset trajectory of PTSD | 93.33% |
| McFarlane, 1987  Extreme bushfire  Australia  (45) | Cross-sectional  4, 11, 29months | *n*=469  Undisclosed  Undisclosed  35.1  Firefighters | Psychological morbidity (*GHQ*) | Age  Social class  **History of trauma**, similar: associated with higher psychological morbidity  **History of life stressors**: associated with higher psychological morbidity | 86.67% |
| Misra et al., 2009 London bombing United Kingdom  (32) | Cross-sectional 2months | *n*=341 109:232  Undisclosed  40.2  Ambulance service personnel | PTSD (*TSQ*)  Stress (*5 adjustment disorder symptoms*) | Age  Gender  History of trauma | 80% |
| Musa & Hamid, 2008  Armed conflict  Darfur  (41) | Cross-sectional  During | *n*=53  23:30  20 to 55  31.6  Aid workers | Secondary traumatic stress (*ProQOL*)  Anxiety (*GHQ*)  Depression (*GHQ*)  Somatic symptoms (*GHQ*)  Burnout (*ProQOL; RWBQ*) | **Age**: younger associated with high rates of secondary traumatic stress and burnout  **Gender**: women associated with higher rates of burnout | 68.80% |
| North et al., 2002b  Oklahoma City bombing  United States  (68) | Cross-sectional  34months | *n*=181  5:176  Undisclosed  38.5  Firefighters | PTSD (*DIS*)  Generalised anxiety disorder (*DIS*)  Panic disorder (*DIS*)  Depression (*DIS*)  Alcohol use disorder (*DIS*)  Drug use disorder (*DIS*) | **History of mental illness** (*DIS*): associated with higher PTSD, anxiety related disorder, depression, alcohol and drug use disorder morbidity; associated with higher morbidity of non-alcoholic disorders four-fold; history of PTSD associated with higher PTSD morbidity post-disaster | 93.33% |
| Pietrzak et al., 2014  World Trade Centre  United States  (54) | Cross-sectional, 3 waves  3, 6, 8years | *n*=10,835  Police: 592:3,443 Non-traditional: 913:5,886  Police: 41.2  Non-traditional: 45.3  Undisclosed  Police (*n*=4,035);  Non-traditional responders (*n*=6,800) | PTSD (*PCL-S*) | *Police : non-traditional responders* **Age**: older associated with a higher severe chronic trajectory of PTSD : older associated with a higher severe chronic trajectory of PTSD and younger associated with a higher improving trajectory of PTSD  **Gender**: women associated with a higher delayed-onset trajectory of PTSD : women associated with a higher severe and moderate chronic trajectory of PTSD, but also an improving trajectory  **Ethnicity**: Hispanic associated with a higher severe chronic trajectory of PTSD : Hispanic associated with a higher severe chronic, delayed-onset, subsyndromal increasing, moderate chronic and improving trajectory of PTSD  **Education**: lower education associated with a higher delayed-onset trajectory of PTSD : lower education associated with a higher severe chronic, delayed-onset, subsyndromal increasing, moderate chronic and improving trajectory of PTSD  **Marital status**: : widowed, separated or divorced associated with a higher severe chronic and delayed onset trajectory of PTSD; married/cohabiting associated with a higher moderate chronic trajectory of PTSD  **Income**: higher associated with a higher severe chronic and delayed onset trajectory of PTSD  **History of mental illness** (depression, anxiety, PTSD): associated with a higher severe chronic, delayed-onset or improving trajectory of PTSD : associated with a higher severe chronic, delayed-onset, subsyndromal increasing, moderate chronic and an improving trajectory of PTSD  **History of life stressors**: : associated with a higher severe chronic, delayed-onset, subsyndromal increasing, moderate chronic and an improving trajectory of PTSD | 100% |
| Pietrzak et al., 2012  World Trade Centre  United States  (52) | Cross-sectional  0.8 to 7years, 4years (average) | *n*=8,466  Undisclosed  Undisclosed  Undisclosed  Police | PTSD (*PCL-S*) | **Age**: older associated with higher PTSD morbidity  **Gender**: women associated with higher PTSD morbidity  **Ethnicity**: Hispanic and ‘Other’ associated with higher PTSD morbidity  **Education**: < high school degree associated with higher PTSD morbidity  **Marital status**: widowed, separated, divorced associated with higher PTSD morbidity  **Income**: lower associated with higher PTSD morbidity  **History of life stressors**: associated with higher PTSD morbidity | 80% |
| Razik et al., 2013  Terrorist attacks  Pakistan  (33) | Cross-sectional  Undisclosed | *n*=125  0:125  Undisclosed  27.87  Emergency relief workers | PTSD (*IES-R*) Anxiety (*PA&D*)  Depression (*PA&D*) Somatic symptoms (*BSI^1^*) | Age  **Marital status**: unmarried associated with higher rates of depression and somatic symptoms  **History of trauma** (*PTS*): associated with higher PTSD, anxiety, and depression morbidity, and higher rates of somatic symptoms | 73.33% |
| Sim & Chua, 2004  SARS outbreak  Singapore  (46) | Cross-sectional  3months | *n*=277 (nurses *n*=186; doctors *n*=91)  Nurses: 185:1  Doctors: 104:82  Undisclosed  Nurses: 40.7  Doctors: 35.2  Nurses and doctors | PTSD (*IES-R*)  Psychological morbidity (*GHQ-28*) | **Age**: younger associated with higher PTSD morbidity  **Marital status**: married associated with higher PTSD morbidity | 73.33% |
| Spinhoven & Verschuur, 2006  Bijlmermeer aviation disaster  Netherlands  (47) | Cross-sectional, 2 waves  11, 13-28months | *n*=1951  150:1801  Undisclosed  45  Disaster workers (police, firefighters, accident and wreckage investigators) | Psychological distress (*GHQ*)  Fatigue (*CIS*)  Quality of life (*EQ-5D*) | **Age**: younger associated with higher rates of fatigue  **Gender**: women associated with higher rates of fatigue  Ethnicity  Education  **Reassured by physicians** (*RQ*): not reassured associated with higher rates of fatigue, persistent fatigue, psychological distress and lower quality of life | 100% |
| Stellman et al., 2008  World Trade Centre  United States  (42) | Cross-sectional  10-61months | *n*=10,132  1,285:8,847  Undisclosed  42.1 ± 9.1  Relief, recovery, clean-up, recovery workers | PTSD (*PCL*)  Depression (*PHQ*)  Panic disorder (*PHQ*) | **Age**: older associated with higher PTSD and depression morbidity  **Gender**: women associated with higher depression and panic morbidity  **Ethnicity**: Hispanic ethnicity associated with higher PTSD, depression, and panic morbidity  **Education**: < high school associated with higher PTSD, depression, and panic morbidity  **Marital status**: separated, divorced, widowed associated with higher PTSD, depression, and panic morbidity | 86.67% |
| Thormar et al., 2013  Earthquake  Indonesia  (34) | Cross-sectional, 3 waved  6, 12, 18months | *n*=506  132:374  Undisclosed  < 30  Red Cross volunteers | PTSD (*IES*)  Anxiety (*HADS*)  Depression (*HADS*) | **Age**: younger associated with higher rates of anxiety  **Gender**: men associated with higher rates of depression  Experience as a disaster relief worker | 86.67% |
| Tosone et al., 2011  World Trade Centre  United States  (35) | Cross-sectional  6years | *n*=481  385:97  60  Undisclosed  Social workers in direct mental health practice | PTSD (*PCL*)  Secondary traumatic stress (STS)  Psychological distress (*Question*)  Burnout (CFS/STS)  Resilience (*CDRS*) | Age  Gender  Ethnicity  Education  Income  **History of trauma** (*LEC*): associated with higher rates of resilience and secondary traumatic stress  **Attachment style** (*AAQ*): avoidant and ambivalent attachment associated with lower rates of resilience and higher rates of secondary traumatic stress | 80% |
| Tucker et al., 2002  Oklahoma City 1995 bombing  United States  (36) | Cross-sectional retrospective  2years assessing 1year  post-deployment symptoms | *n*=51  16:35  25 to 56  34.7  Body handlers | PTSD (*DIS*)  Depression (*GI*) | Age  Gender  History of trauma  Experience as a disaster relief worker | 80% |
| Ursano et al., 1995  USS *Iowa* gun turret explosion  Puerto Rico  (37) | Cross-sectional, 3 waves  1, 4, 13months | *n*=54  5:49  19 to 48  29  Volunteer body handlers | PTSD (*IES; SC-90-R; 12 items)*  Anxiety (*SC-90-R*)  Depression (*ZDS; SC-90-R*)  Somatic symptoms (*SC-90-R*)  Hostility (*SC-90-R*) | Age  **Ethnicity**: at 1month, white ethnicity associated with higher rates of somatic symptoms  Education  **Marital status**: at 1 and 4months, being single was associated with higher PTSD-avoidance morbidity  Experience as a disaster relief worker | 80% |
| Van der Velden et al., 2012  Haiti earthquake disaster  Netherlands  (77) | Prospective, undisclosed time pre-deployment to 3months post | *n*=51  4:47  Undisclosed  44.7  Police (*n*=11); Firefighters (*n*=35); Ambulance staff (4); Surgeon (n=1) | PTSD (*IES*)  Anxiety (*SC-90-R*)  Depression (*SC-90-R*) | Experience as a disaster relief worker | 86.67% |
| Van der Velden et al., 2008  Enschede fireworks disaster  Netherlands  (38) | Cross-sectional, 2 waves  2-3weeks, 18months | *n*=66  21:45  Undisclosed  38.2  Ambulance personnel | PTSD (*IES*)  Depression (*SC-90-R*)  Hostility (*SC-90-R*) | Age  Gender  **Education**: associated with higher rates of depression, education level undisclosed | 86.67% |
| Wang et al., 2011  Wechuan earthquake  China  (39) | Cross-sectional  6months | *n*=1,056  0:1,056  18 to 32  21  Military first responders | PTSD (*DTS-C*) | Age  **Gender**: women associated with higher PTSD morbidity  Education (< college)  Marital status  **Childhood environment** (rural vs. urban / **siblings** / **parents separated**): single child associated with higher PTSD morbidity; parents separated associated with higher PTSD morbidity | 93.33% |
| Weiss et al., 1995  Interstate-880 Freeway Collapse  United States  (75) | Cross-sectional  Undisclosed | *n*=154  19:135  Undisclosed  38.5  Emergency medical personnel, road workers, maintenance, police, firefighters | PTSD (*IES-R; M-PTSD*)  Psychological distress (*S-90 GSI*) | **Experience as a disaster** **worker**: less associated with higher rates of PTSD and psychological distress  **Personality traits** (adjustment) (*HPI, Adjustment subscale*): associated with lower rates of PTSD and psychological distress  **Locus of control** (*LOC*): external associated with higher rates of PTSD and psychological distress | 93.33% |
| Wu et al., 2009  SARS outbreak  China  (58) | Cross-sectional  3years | *n*=549  412:137  Undisclosed  36-50  Hospital employees | PTSD (*IES-R*) | **Age**: < 50 associated with higher rates of PTSD  Gender  Education  Marital status  Income  History of trauma | 86.67% |
| Zhen et al., 2012  Wenchan earthquake  China  (59) | Cross-sectional  Within 1year | *n*=210  210:0  23 to 40  Undisclosed  Red Cross Nurses | PTSD (*TSS-C*) | **Age**: younger associated with higher PTSD morbidity  Education  Marital status  **History of mental illness**: associated with higher PTSD morbidity  **Experience as a disaster** **worker**: less associated with higher PTSD morbidity | 86.67% |
